# Ascites—VIII

**Published:** 1909-07-31

**Authors:** 


### Medicine.

### ASCITES—VIII.

### THE DIFFERENTIAL DIAGNOSIS OF THE CAUSE OF ASCITES (*Continued*).

*Cirrhosis of the Liver.*—If ascites is due to this cause there is likely to be a history of former chronic alcoholism, even though the patient may have been latterly teetotal. One would expect to find symptoms and signs of alcoholic habits, such as nausea or sickness in the morning, loss of appetite for breakfast, a desire for a mid-morning dram, a history of epistaxis, hæmatemesis, or melæna, acne rosacea, or dilated stellate venules on the face and around the costal margin. It may be possible to feel the liver edge firm and roughened; the spleen may also be enlarged, although in adults suffering from hepatic cirrhosis it rarely extends as far down as the level of the umbilicus. There is a variety of cirrhosis in young persons, on the other hand, designated "splenomegalic" on account of the striking enlargement of the spleen that occurs in it. Urobilin and bile salts may be detected in the urine, even when jaundice is absent.

As a rule the occurrence of ascites in cirrhosis is a late sign, and the patient seldom lives many months after it has become necessary to tap the abdomen for it. Hale White lays much stress upon this as an aid to diagnosis—an ascites may be diagnosed as due to cirrhosis of the liver, but if the patient survives to have four or five tappings, or more, the original diagnosis is likely to be wrong, and it should nearly always be revised to perihepatitis.

*Perihepatitis.*—Ascites may be the only indication of perihepatitis, and unless the liver can be palpated the diagnosis is most uncertain. An ascites that was originally due to some other cause may, as the result of repeated tappings, become continued by the supervention of perihepatitis, even though the original cause of ascites becomes cured. Besides these cases, however, there are others in which perihepatitis antedates the first tapping. The condition is, in a sense, only a variety of chronic simple peritonitis.

The capsule of the liver becomes opaque, white, and very much thickened; it contracts and distorts the shape of the organ, especially its edge, which becomes either very blunt, or else curled up, under the main mass of the viscus. The surface is frequently covered with numbers of pits, which give it an appearance that has been compared to that of skin that is scarred as the result of smallpox.

If the turned-up, or folded under, condition of the edge of the liver can be determined by palpation, it is pathognomonic of perihepatitis. Albuminuria occurs in the majority of the cases of this disease. Hale White found on analysing twenty-two cases of universal perihepatitis that the kidneys were pathologically granular in nineteen.

It is often very difficult to distinguish between cirrhosis of the liver with ascites and perihepatitis. Jaundice very rarely occurs in the latter, whereas it is not uncommon in cirrhosis. If albuminuria is present it is in favour of a diagnosis of perihepatitis, for although alcoholism is a responsible cause both of cirrhosis of the liver and of granular kidney, it is decidedly rare to find the two lesions well marked in one and the same case.

*Syphilis.*—Ascites is not often caused by syphilis. There are three ways, however, in which it may possibly be so caused: As the result of intercellular cirrhosis of the liver due to congenital syphilis, as perihepatitis, and by a gumma, or the scar of a healed gumma, stenosing the portal vein.

In cases of ascites with enlargement of the liver, the possibility of syphilis being at the root of the mischief should not be forgotten, on account of its important bearing on the treatment of the case. A history of a primary lesion, followed by a sore throat, or a rash, may be inquired for, and later signs, such as nodes or ulcers, may be looked for.

The liver in this condition may be greatly enlarged, and sometimes it can be felt to contain large and irregular masses, which may easily be mistaken for malignant disease or even for abscesses. The lumps in such a case are gummatous; the healing of a gumma does not restore the surface of the liver to its original smooth condition, for where each gumma has been there will be a permanent, deeply-depressed scar, which may easily be mistaken for an umbilicated mass of secondary growth.

*Carcinoma of the Liver.*—Ascites due to carcinoma of the liver is usually associated with marked and rapid emaciation, cachexia, and often intense and persistent jaundice. In a typical case the liver is considerably enlarged, and if there is not a very great amount of fluid in the peritoneal cavity, it may be felt extending to the level of the umbilicus, or even below it.

The edge may be determined to be hard, thickened, irregular, and coarsely nodular, the surface to be hard, tender, and very irregular from large projecting nodules of growth, the central part of which may be umbilicated. In some cases in which the presence of decided nodules cannot be determined, it may be difficult to distinguish between cirrhosis and carcinoma. A careful investigation should always be made for signs or symptoms of a primary growth, especially of the stomach, rectum, gall-bladder, and pancreas. Rectal examination should on no account be omitted. It is not common for cirrhosis of the liver to cause intense jaundice simultaneously with ascites, so that if the jaundice is well marked it favours the diagnosis of carcinoma. Wasting and weakness occur with cirrhosis as well as with carcinoma, so that too much stress must not be laid on these symptoms in the differential diagnosis. Moderate intermitting pyrexia may also be associated with both diseases.

In cases of primary diffuse carcinoma of the liver, a correct diagnosis may be impossible, and even at a post-mortem examination it may be difficult to express a definite opinion until a histological examination has been made.

(*To be continued.*)

